# Launching *Clinical and Public Health Guidelines*: A unique journal for the guidelines field

**DOI:** 10.1002/gin2.12009

**Published:** 2024-02-15

**Authors:** Ivan D. Florez

**Affiliations:** ^1^ Department of Pediatrics Universidad de Antioquia Medellin Colombia; ^2^ School of Rehabilitation Science McMaster University Hamilton Canada; ^3^ Pediatric Intensive Care Unit Clinica Las Américas‐AUNA Medellin Colombia

The first traceable guidelines in the literature were consensus statements (i.e., recommendations formulated during specialties conferences in the United States) developed by professional associations and based on the participants' experience and opinions. This methodology has been called GOBSAT (Good Old Boys Sat Around the Table), namely, the process by which mainly self‐selected experts discuss their (often subjective) opinions and provide recommendations.^1^ During the early 1990s, the evidence‐based medicine (EBM) movement was born. Clinical practice guidelines gradually started implementing this approach and shifted from the expert‐based approach to a process based on systematic reviews to answer clinically relevant questions.^2^ As a result, ‘systematic review’ was incorporated in the definition by, formerly, the United States Institute of Medicine (currently, the National Academy of Health) in 2011: guidelines are ‘statements that include recommendations, intended to optimize patient care, that are informed by a systematic review of evidence and an assessment of the benefits and harms of alternative care options’.^3^ This newer generation of evidence‐based guidelines quickly became the ideal methodological approach to develop more transparent recommendations. However, the evolution of guidelines was far from ending.

Additional factors were also identified as key to informing the development of recommendations. The consideration of patients' values and preferences, costs and use of resources, the impact on health equity and the feasibility and applicability of the recommended actions have been identified as critical in the recommendation process. In short, guidelines need to provide recommendations supported by evidence but also feasible, affordable, usable and acceptable to users and patients. Therefore, guidelines evolved from consensus‐based statements to recommendations that use evidence‐based methods to work with research evidence and, later, to systematically consider patients' values and preferences, costs and resources used and the feasibility of the recommendations.^4^

This exciting evolution of guidelines has been possible thanks to the work of many guidelines developers, researchers and users who have contributed for decades to improve guidelines in different ways. It is impossible to list all the key actors that have contributed, but perhaps the most significant milestones in this journey are the emergence of the EBM movement, the development and expansion of systematic review methods, the development of the AGREE tool,^5^ the establishment of the Guidelines International Network (GIN)^6^ and the constitution of the Grading of Recommendations, Assessment, Development and Evaluations working group.^7^

As a paediatrician, I am a guidelines user. However, my relationship with guidelines is much closer and gratifying. I have been a guidelines chair, methodologist, implementer, panellist and guideline and evidence synthesis researcher. I have spent 15 years developing guidelines, leading a guideline programme, training or teaching about guidelines or researching on guidelines methods. At many levels and contexts, I have witnessed how guidelines are an extraordinary tool to reduce the gap between science and clinical practice or policy making. Surprisingly, the guidelines field did not have a specific journal where users, researchers and developers could put their products, start an academic discussion about guidelines and facilitate recommendations dissemination.

In response to this gap, we are launching *Clinical and Public Health Guidelines*, the first scientific journal exclusively focused on guidelines. *Clinical and Public Health Guidelines* is open‐access, and as such, all its articles are universally accessible online. Our journal considers two types of manuscripts: those related to the science of guidelines, and guidelines. By manuscripts related to the science of guidelines, we mean primary and secondary research aiming to understand and improve any aspect of guidelines development and use, the protocols of those types of articles and methodological manuscripts discussing guidelines development challenges or the development and application of methodological tools.

Regarding the guidelines' manuscripts, we are considering clinical or public health evidence‐based guidelines or health systems guidance. These include full, adapted, de novo or updated guidelines, public versions of guidelines and living guidelines. Lastly, we are also considering guidelines protocols. The establishment of guidelines registration as a major step towards more transparency and collaboration should facilitate the development of more guidelines protocols. We expect *Clinical and Public Health Guidelines* will be the home of many of them.

There are many challenges ahead of us. Urgent topics will be covered in these pages in the following months, years and decades, and will help shape the field of guidelines field. Some of them are the role of artificial intelligence and real‐world evidence in guidelines, rapid and living recommendations development and implementation, guidelines registration, patients and citizen versions of guidelines, research integrity at the evidence synthesis and guideline levels, guidelines development in low‐ and middle‐income countries, guidelines adaptation, implementation of recommendations, the guidelines roles in legal issues, guidelines training and certification, and many more.

I feel privileged, humbled and grateful to take the leadership of this journal. Special thanks to the GIN Board of Trustees for this opportunity and to Zachary Munn, who was the architect behind the birth of the journal, and who wrote an editorial for this inaugural issue.^7^ We have gathered an unparalleled team of global guidelines experts to support the journal as Editorial Board members or Associate Editors. I am very grateful to this group of colleagues and friends who, despite their busy schedules, accepted our invitation to join this journey. I hope our teamwork will translate into success for the Journal in the next few years.

Previously, I mentioned some milestones in the guidelines world. We should now add *Clinical and Public Health Guidelines* to that list. Having our own journal is, without doubt, another step towards recognizing the importance of guidelines in health care and public health. Therefore, I am confident that the launching of *Clinical and Public Health Guidelines* will be an inflection point in our aim to disseminate the science of guidelines among all the users, developers and researchers, which in turn will help to reduce the gap between the knowledge and action and improve the health care and policy decision/making process.

## CONFLICT OF INTEREST STATEMENT

The author is the current lead of the AGREE collaboration and is a member of the GRADE working group.
